# Supplementary material for: Medication Regimen Complexity Index Score at Admission as a Predictor of Inpatient Outcomes: A Machine Learning Approach
Source: Int J Environ Res Public Health. 2023 Feb 20;20(4):3760. doi: 10.3390/ijerph20043760 (PMC9967355; doi:10.3390/ijerph20043760)
Supplement: Supplementary file 1 [file ijerph-20-03760-s001.zip › Supplemental S1.docx]

**Table S1:** MRCI calculation algorithm [28].


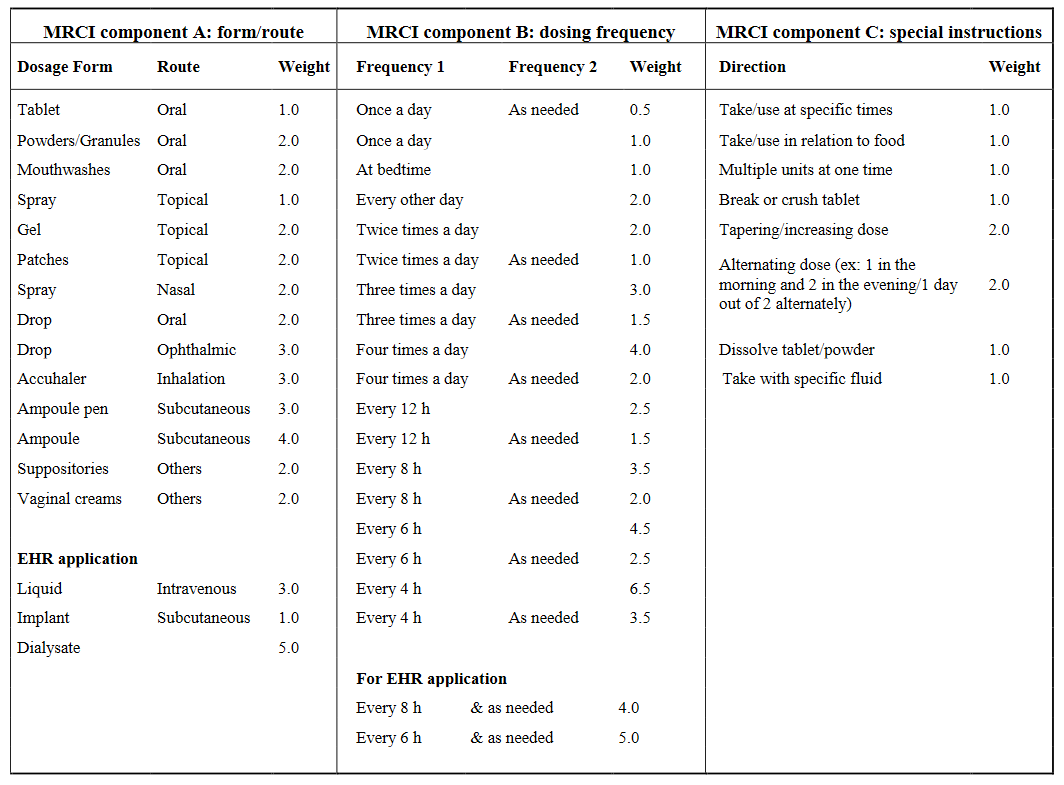


**Table S2**: Model specifications for prediction models for inpatient mortality, length of stay (LOS), and need for mechanical ventilation (MV).

| **Prediction models** | **Input variables** | **Output of interests** |
| --- | --- | --- |
| Home Medication Model (MRCI) | Age, Race, Gender, Charlson score, Health insurance, BMI, and MRCI | 1.  Inpatient mortality        0: survived and discharge        1: expired    2. Length of stay       0: <72 hours,       1: >=72 hours    3. Need for mechanical ventilation       0: no MV       1: on MV |
| Admission Model (APACHEII) | Age, Race, Gender, Charlson score, Health insurance, BMI and, APACHEII |  |
| MRCI & APACHEII Model | Age, Race, Gender, Charlson score, Health insurance, BMI, MRCI, and APACHEII |  |
| SOFA Model | Age, Race, Gender, Charlson score, Health insurance, BMI, and SOFA_24hours |  |
| MRCI & SOFA Model | Age, Race, Gender, Charlson score, Health insurance, BMI, MRCI, and SOFA_24hours |  |

Abbreviations: MRCI: Medication Regimen Complexity Index , APACHEII: The Acute Physiology and Chronic Health Evaluation score, SOFA: Sequential Organ Failure Assessment score; MV: Mechanical ventilation.
