# Supplementary material for: Medication Regimen Complexity Index Score at Admission as a Predictor of Inpatient Outcomes: A Machine Learning Approach
Source: Int J Environ Res Public Health. 2023 Feb 20;20(4):3760. doi: 10.3390/ijerph20043760 (PMC9967355; doi:10.3390/ijerph20043760)

**Figure S1: Number of Medications at home according to MRCI group**


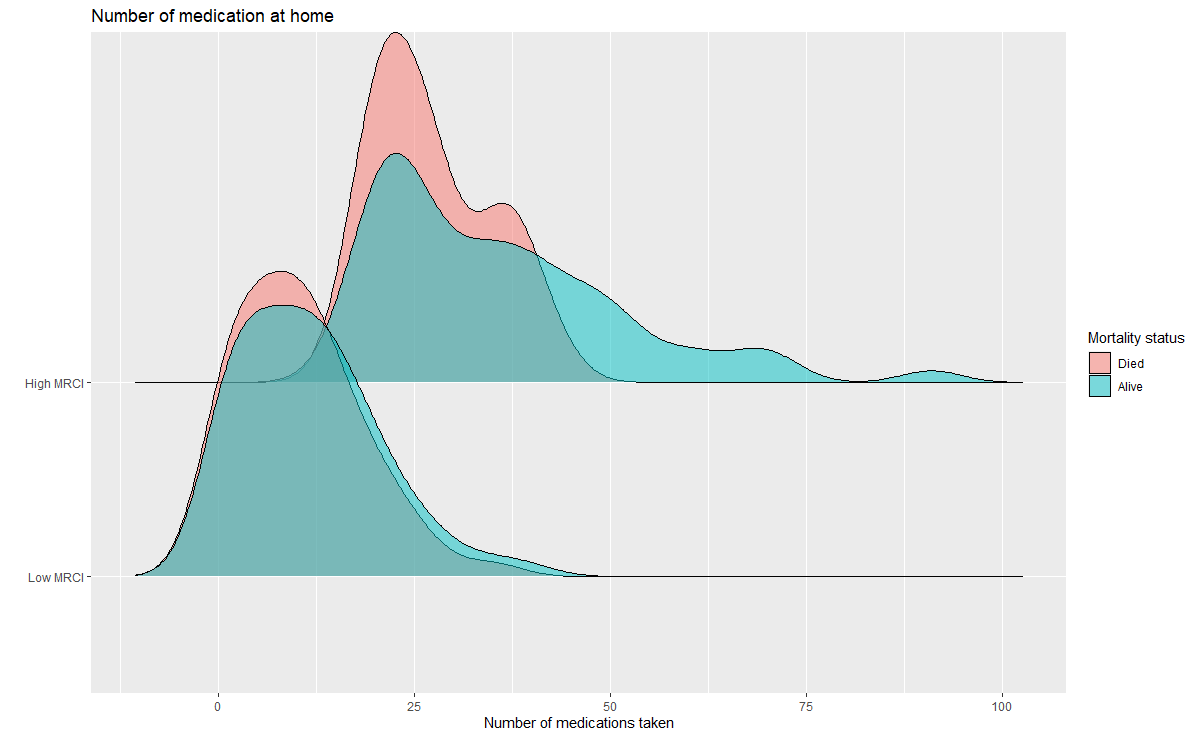


**Figure S2: Percentage of dead or alive patients based on LOS and MRCI**


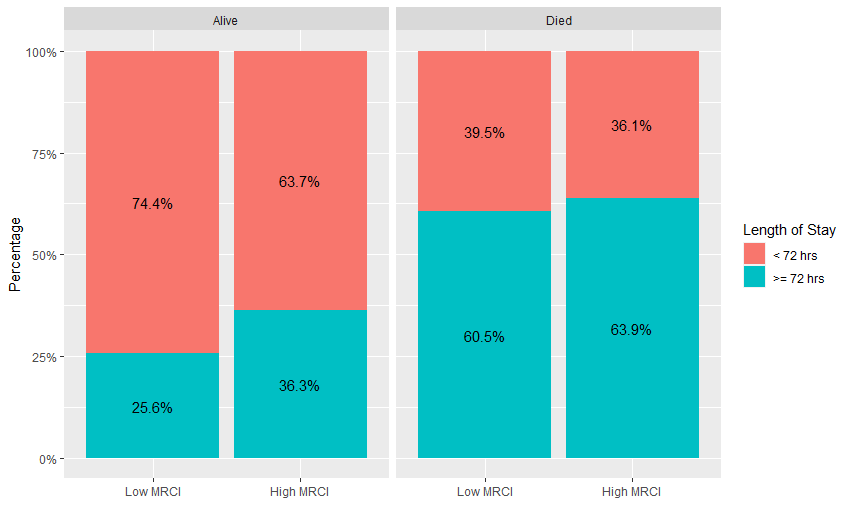


**Figure S3: Percentage of dead or alive patients based on MVB and MRCI**


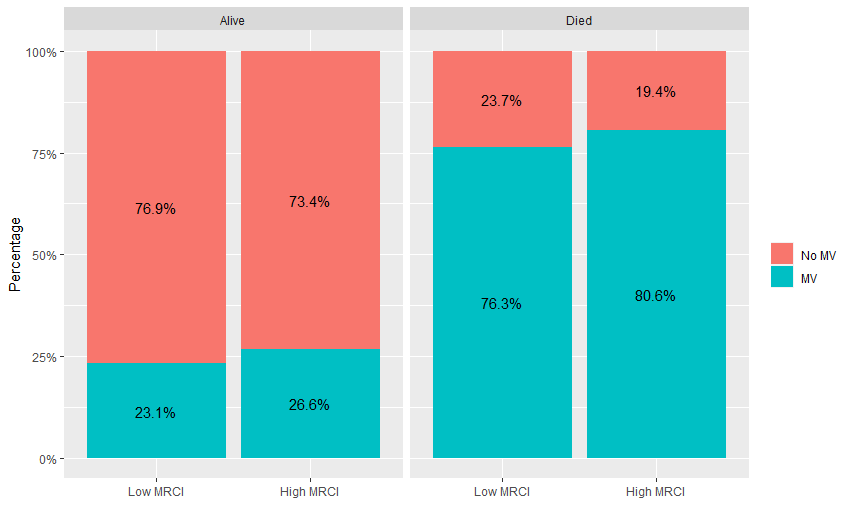


**Figure S4: Number of medications taken the day of admission**

**
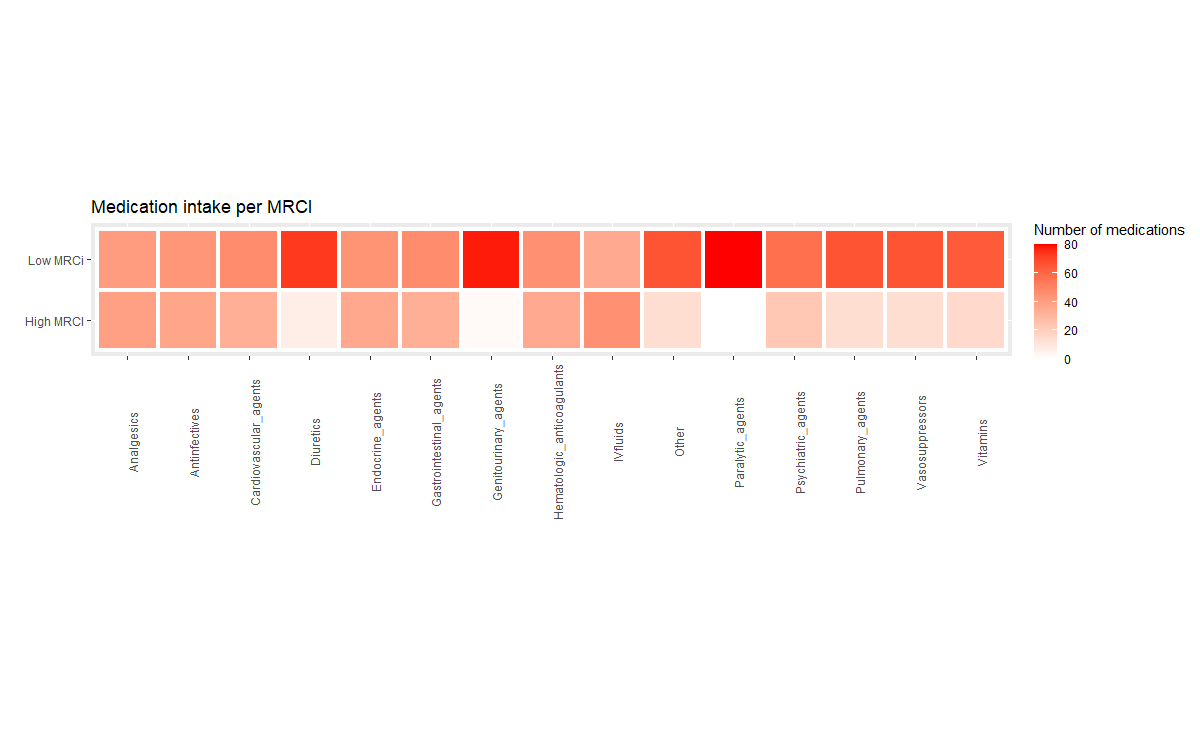
**

**Figure S5:** **Distribution of race according to MRCI thresholds**

**
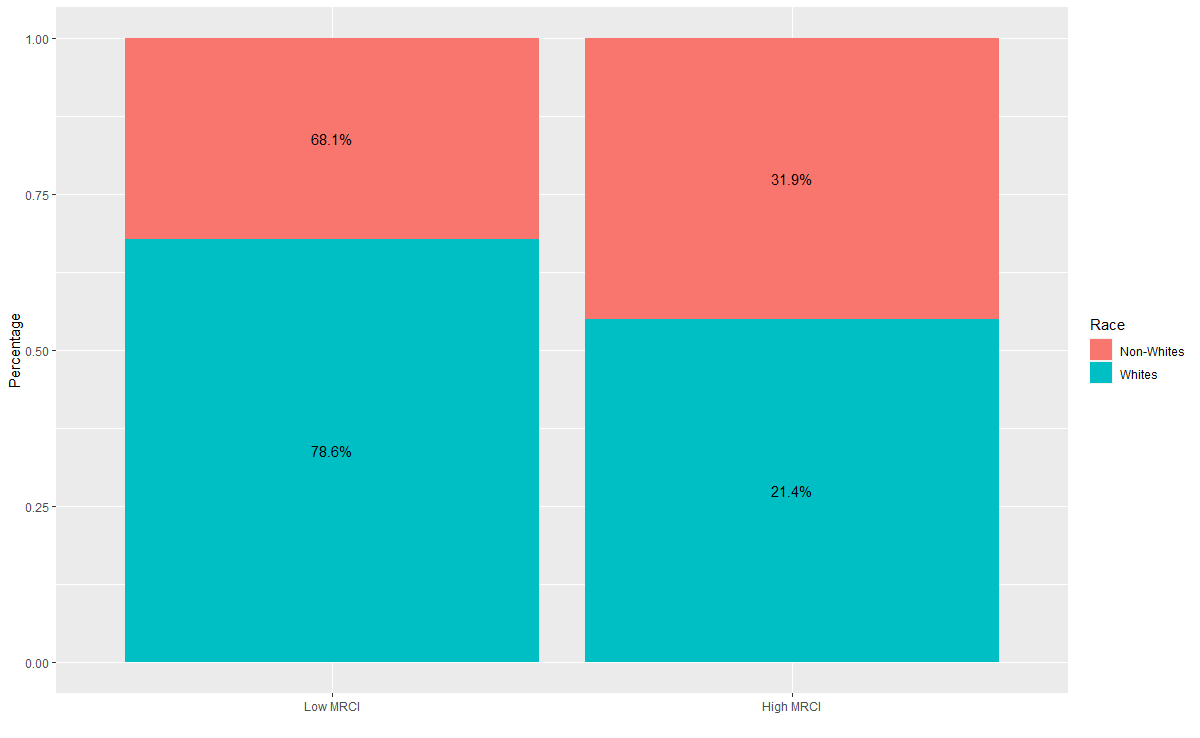
**

**Predisposing factors**

**Figure S6:** **Distribution of Hypertension according to MRCI thresholds**


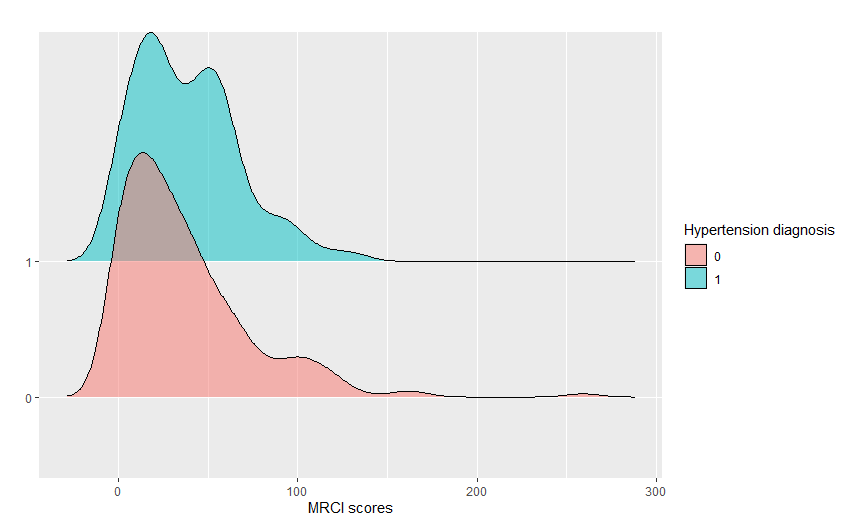


**Figure S7:** **Distribution of acute renal failure according to MRCI thresholds**


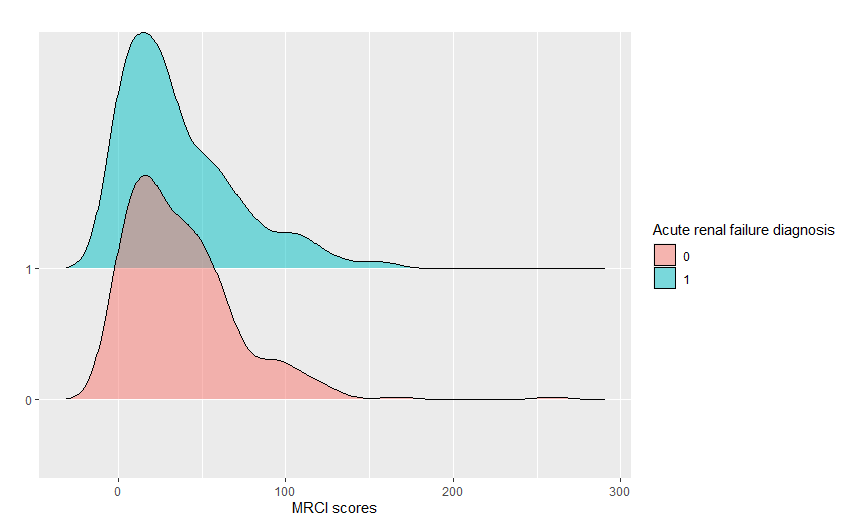


**Figure S8:** **Distribution of myocardial infarction according to MRCI thresholds**


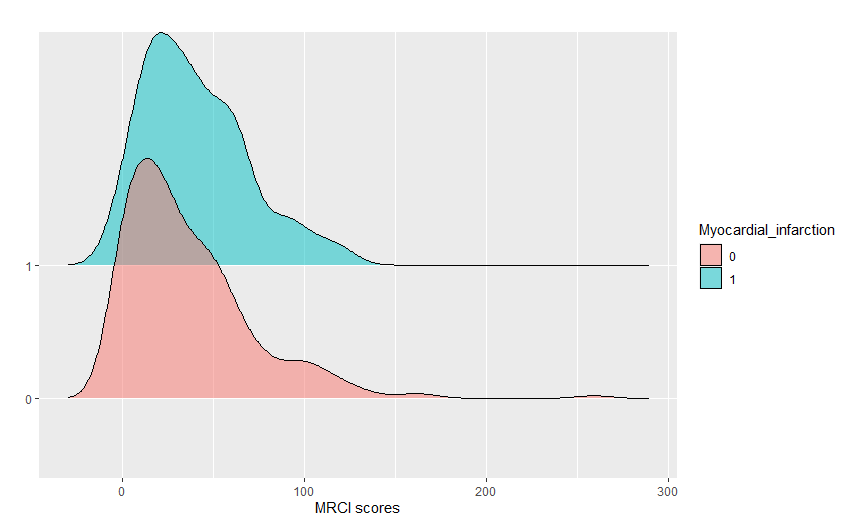


**Figure S9:** **Distribution of metabolic encephalopathy according to MRCI thresholds**


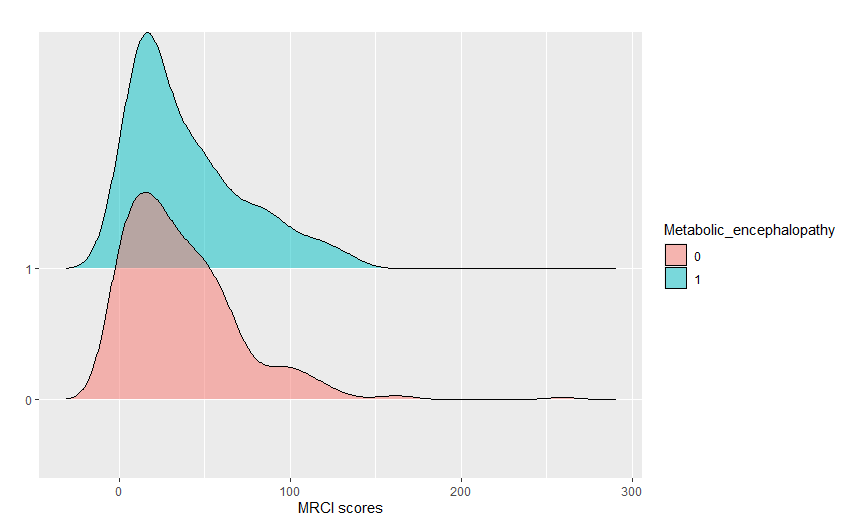


**Figure S10:** **Distribution of long-term insulin use according to MRCI thresholds**


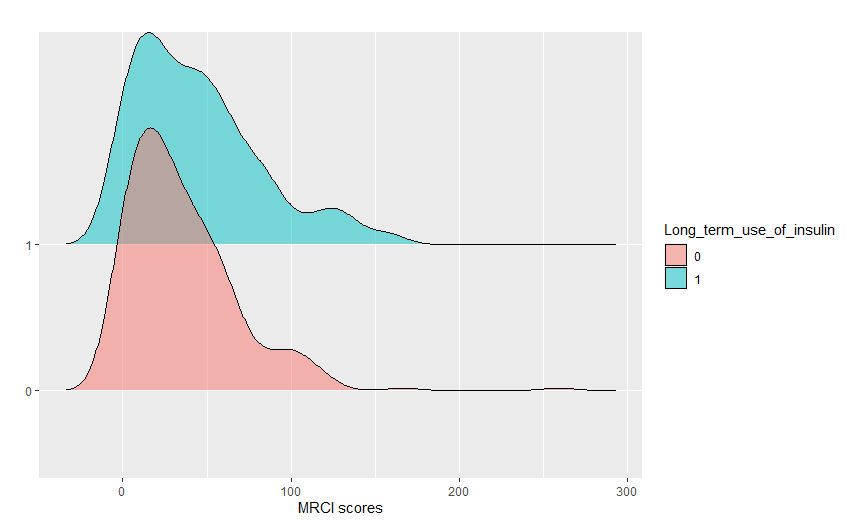

Supplement: Supplementary file 1 [file ijerph-20-03760-s001.zip › Supplemental S2.docx]
