# Supplementary material for: Medication Regimen Complexity Index Score at Admission as a Predictor of Inpatient Outcomes: A Machine Learning Approach
Source: Int J Environ Res Public Health. 2023 Feb 20;20(4):3760. doi: 10.3390/ijerph20043760 (PMC9967355; doi:10.3390/ijerph20043760)
Supplement: Supplementary file 1 [file ijerph-20-03760-s001.zip › Supplemental S3.docx]

| **Table S3: Performance of all machine learning algorithms across the entire population and across subgroups** | | | | | | | | | | | | | | | |
| --- | --- | --- | --- | --- | --- | --- | --- | --- | --- | --- | --- | --- | --- | --- | --- |
|  | **AUC** | | | **Sensitivity** | | | **Specificity** | | | **PPV** | | | **NPV** | | |
|  | **All** | **White** | **Non-White** | **All** | **White** | **Non-White** | **All** | **White** | **Non-White** | **All** | **White** | **Non-White** | **All** | **White** | **Non-White** |
| **Outcome: Mortality** | | | | | | | | | | | | | | | |
| **MRCI** | | | | | | | | | | | | | | | |
| **LRG** | 0.47  (0.42 – 0.51) | 0.55  (0.51 – 0.59) | 0.67  (0.63 -0.71) | 0.50  (0.45 -0.55) | 0.42  (0.38 – 0.47) | 0.56  (0.51 -0.61) | 0.47  (0.41 -0.53) | 0.37  (0.31 – 0.44) | 0.63  (0.58 – 0.68) | 0.57  (0.52 -0.62) | 0.55  (0.50 -0.60) | 0.59  (0.54 – 0.65) | 0.40  (0.35 -0.46) | 0.26  (0.22 -0.31) | 0.59  (0.54 -0.64) |
| **NB** | 0.54  (0.50 – 0.58) | 0.50  (0.45 -0.54) | 0.58  (0.54 -0.62) | 0.53  (0.48 -0.58) | 0.52  (0.47 -0.57) | 0.44  (0.40 -0.49) | 0.51  (0.45 – 0.57) | 0.54  (0.49 – 0.60) | 0.48  (0.42 -0.55) | 0.59  (0.53 – 0.64) | 0.58  (0.53 – 0.63) | 0.66  (0.60 - 0.71) | 0.46  (0.40 – 0.51) | 0.49  (0.43 – 0.54) | 0.28  (0.24 -0.33) |
| **RF** | 0.60  (0.56 -0.64) | 0.55  (0.51 – 0.59) | 0.45  (0.41 - 0.49) | 0.56  (0.51 -0.60) | 0.49  (0.45 – 0.52) | 0.40  (0.36 -0.45) | 0.69  (0.60 – 0.77) | 0.00  (0.00 – 0.26) | 0.27  (0.20 – 0.35) | 0.89  (0.86 -0.92) | 0.97  (0.94 – 0.96) | 0.70  (0.65 -0.75) | 0.24  (0.20 -0.29) | 0.00  (0.00 – 0.01) | 0.10  (0.07 -0.13) |
| **XGB** | 0.69  (0.66 -0.73 | 0.81  (0.78 – 0.84) | 0.73  (0.70 – 0.75) | 0.73  (0.68 -0.78) | 0.83  (0.79 – 0.87) | 1.00  (0.98 – 1.00) | 0.67  (0.63 -0.72) | 0.79  (0.75 – 0.83) | 0.61  (0.57 -0.65) | 0.59  (0.54 -0.64) | 0.78  (0.74 – 0.82) | 0.45  (0.40 -0.50) | 0.80  (0.75 -0.84) | 0.84  (0.79 – 0.87) | 1.00  (0.99 – 1.00) |
| **APACHEII** | | | | | | | | | | | | | | | |
| **LRG** | 0.86  (0.83 – 0.89) | 0.78  (0.75 – 0.82) | 0.80  (0.77 – 0.83) | 0.86  (0.81 – 0.89) | 0.62  (0.58 – 0.67) | 0.70  (0.65 – 0.75) | 0.72  (0.67 – 0.76) | 0.75  (0.69 – 0.80) | 0.83  (0.78 – 0.87) | 0.68  (0.63 – 0.72) | 0.82  (0.78 – 0.86) | 0.84  (0.79 – 0.88) | 0.88  (0.84 – 0.91) | 0.51  (0.46 – 0.57) | 0.69  (0.64 – 0.73) |
| **NB** | 0.86  (0.83 – 0.89) | 0.80  (0.77 – 0.84) | 0.50  (0.45 – 0.54) | 0.81  (0.76 – 0.85) | 0.86  (0.81 – 0.90) | 0.44  (0.40 – 0.49) | 0.73  (0.69 – 0.78) | 0.73  (0.68 – 0.77) | 0.48  (0.42 – 0.55) | 0.72  (0.67 – 0.76) | 0.66  (0.61 – 0.71) | 0.66  (0.60 – 0.71) | 0.82  (0.77 – 0.86) | 0.89  (0.86 – 0.92) | 0.28  (0.24 – 0.33) |
| **RF** | 0.82  (0.78 – 0.85) | 0.73  (0.69 – 0.76) | 0.71  (0.67 – 0.74) | 0.61  (0.57 – 0.65) | 0.58  (0.53 – 0.62) | 0.51  (0.47 – 0.54) | 0.82  (0.75 – 0.87) | 0.72  (0.65 – 0.78) | 0.86  (0.76 – 0.93) | 0.92  (0.89 – 0.940 | 0.85  (0.81 – 0.88) | 0.97  (0.94 – 0.98) | 0.38  (0.33 – 0.43) | 0.39  (0.34 – 0.44) | 0.18  (0.14 – 0.22) |
| **XGB** | 0.91  (0.89 – 0.93) | 0.76  (0.73 – 0.79) | 0.74  (0.71 – 0.76) | 0.88  (0.84 – 0.91) | 0.78  (0.73 – 0.82) | 0.95  (0.91 – 0.97) | 0.94  (0.91 -0.96) | 0.74  (0.70 – 0.79) | 0.63  (0.59 – 0.67) | 0.94  (0.91 – 0.96) | 0.74  (0.69 – 0.78) | 0.51  (0.46 – 0.56) | 0.87  (0.83 – 0.91) | 0.79  (0.74 – 0.83) | 0.97  (0.94 – 0.98) |
| **MRCI&APACHEII** | | | | | | | | | | | | | | | |
| **LRG** | 0.85  (0.82 – 0.88) | 0.85  (0.81 – 0.87) | 0.78  (0.75 – 0.82) | 0.87  (0.82 -0.90) | 0.68  (0.63 – 0.72) | 0.76  (0.72 – 0.81) | 0.76  (0.71 – 0.80) | 0.80  (0.75 – 0.85) | 0.85  (0.80 – 0.88) | 0.74  (0.69 -0.78) | 0.85  (0.81 – 0.88) | 0.84  (0.79 – 0.88) | 0.88  (0.84 – 0.91) | 0.61  (0.56 – 0.66) | 0.77  (0.73 – 0.81) |
| **NB** | 0.86  (0.83 -0.88) | 0.73  (0.69 – 0.77) | 0.54  (0.50 – 0.59) | 0.81  (0.77 -0.85) | 0.73  (0.68 – 0.77) | 0.41  (0.37 – 0.45) | 0.75  (0.71 -0.80) | 0.75  (0.70 – 0.79) | 0.37  (0.30 – 0.45) | 0.75  (0.70 -0.79) | 0.75  (0.70 – 0.79) | 0.66  (0.60 – 0.71) | 0.82  (0.77 – 0.86) | 0.73  (0.68 – 0.77) | 0.18  (0.14 – 0.22) |
| **RF** | 0.83  (0.80 – 0.86) | 0.76  (0.72 – 0.79) | 0.67  (0.63 – 0.71) | 0.64  (0.60 -0.68) | 0.58  (0.53 – 0.62) | 0.54  (0.50 – 0.58) | 0.87  (0.82 - 0.92) | 0.76  (0.69 – 0.82) | 0.91  (0.84 – 0.95) | 0.94  (0.91 -0.96) | 0.88  (0.84 – 0.91) | 0.97  (0.94 – 0.98) | 0.45  (0.40 -0.51) | 0.37  (0.32 – 0.42) | 0.28  (0.23 – 0.32) |
| **XGB** | 0.91  (0.89 – 0.93) | 0.81  (0.78 – 0.84) | 0.77  (0.74 – 0.80) | 0.93  (0.90 -0.96) | 0.80  (0.76 – 0.84) | 0.85  (0.81 – 0.89) | 0.89  (0.85 – 0.92) | 0.82  (0.78 – 0.86) | 0.70  (0.65 – 0.74) | 0.88  (0.84 – 0.91) | 0.83  (0.79 – 0.87) | 0.67  (0.62 – 0.72) | 0.94  (0.91 -0.96) | 0.79  (0.74 – 0.83) | 0.87  (0.82 – 0.90) |
| **SOFA** | | | | | | | | | | | | | | | |
| **LRG** | 0.94  (0.92 – 0.96) | 0.74  (0.70 – 0.78) | 0.84  (0.80 – 0.87) | 1.00  (0.99 – 1.00) | 0.72  (0.67 – 0.77) | 0.76  (0.71 -0.80) | 0.83  (0.79 – 0.86) | 0.77  (0.72 – 0.82) | 0.90  (0.86 – 0.93) | 0.80  (0.76 – 0.84) | 0.79  (0.74 – 0.83) | 0.90  (0.86 – 0.93) | 1.00  (0.99 – 1.00) | 0.70  (0.65 – 0.75) | 0.75  (0.70 – 0.79) |
| **NB** | 0.94  (0.92 – 0.96) | 0.75  (0.71 – 0.79) | 0.62  (0.58 – 0.67) | 1.00  (0.99 – 1.00) | 0.75  (0.71 – 0.80) | 0.46  (0.42 -0.50) | 0.85  (0.81 – 0.88) | 0.79  (0.74 – 0.83) | 0.52  (0.45 – 0.59) | 0.83  (0.78 – 0.86) | 0.79  (0.75 – 0.84) | 0.72  (0.67 – 0.77) | 1.00  (0.99 – 1.00) | 0.75  (0.70 – 0.79) | 0.26  (0.22 -0.31) |
| **RF** | 0.92  (0.91 – 0.94) | 0.85  (0.82 – 0.88) | 0.77  (0.73 – 0.81) | 0.69  (0.64 – 0.73) | 0.68  (0.64 – 0.73) | 0.48  (0.44 – 0.52) | 0.85  (0.80 – 0.89) | 0.91  (0.86 – 0.94) | 0.60  (0.50 – 0.69) | 0.91  (0.87 – 0.93) | 0.94  (0.91 – 0.96) | 0.86  (0.81 – 0.89) | 0.56  (0.51 – 0.61) | 0.58  (0.52 – 0.63) | 0.18  (0.15 – 0.23) |
| **XGB** | 0.97  (0.96 – 0.98) | 0.88  (0.85 – 0.90) | 0.79  (0.76 – 0.82) | 0.94  (0.91 – 0.96) | 0.86  (0.82 – 0.89) | 0.92  (0.88 -0.95) | 1.00  (0.99 – 1.00) | 0.90  (0.86 – 0.93) | 0.70  (0.65 – 0.74) | 1.00  (0.99 – 1.00) | 0.91  (0.87 – 0.93) | 0.65  (0.60 – 0.69) | 0.94  (0.91 – 0.96) | 0.85  (0.80 -0.88) | 0.93  (0.90 – 0.96) |
| **MRCI&SOFA** | | | | | | | | | | | | | | | |
| **LRG** | 0.94  (0.92 – 0.96) | 0.75  (0.72 – 0.79) | 0.85  (0.81 – 0.88) | 0.92  (0.88 – 0.95) | 0.73  (0.68 – 0.77) | 0.67  (0.63 – 0.72) | 0.82  (0.78 – 0.86) | 0.80  (0.75 -0.84) | 0.88  (0.83 -0.91) | 0.81  (0.77 – 0.85) | 0.82  (0.77 – 0.86) | 0.90  (0.86 – 0.93) | 0.92  (0.89 – 0.95) | 0.70  (0.65 – 0.750 | 0.62  (0.57 – 0.67) |
| **NB** | 0.92  (0.90 – 0.94) | 0.71  (0.68 – 0.75) | 0.61  (0.57 – 0.66) | 0.80  (0.76 -0.84) | 0.66  (0.61 – 0.70) | 0.44  (0.39 – 0.48) | 0.85  (0.81 – 0.89) | 0.78  (0.72 – 0.82) | 0.47  (0.40 -0.54) | 0.87  (0.83 – 0.91) | 0.83  (0.79 – 0.87) | 0.66  (0.60 -0.71) | 0.77  (0.72 -0.81) | 0.58  (0.53 -0.63) | 0.26  (0.22 – 0.31) |
| **RF** | 0.91  (0.89 -0.93) | 0.76  (0.72 -0.80) | 0.75  (0.71 – 0.79) | 0.66  (0.61 – 0.70) | 0.67  (0.63 – 0.72) | 0.47  (0.43 – 0.51) | 0.91  (0.86 – 0.95) | 0.88  (0.83 - 0.91) | 0.58  (0.44 – 0.72) | 0.96  (0.93 -0.98) | 0.90  (0.86 – 0.93) | 0.93  (0.90 – 0.96) | 0.47  (0.42 – 0.53) | 0.62  (0.57 – 0.67) | 0.08  (0.06 – 0.11) |
| **XGB** | 0.98  (0.97 – 0.99) | 0.88  (0.85 – 0.90) | 0.79  (0.76 – 0.82) | 0.96  (0.93 -0.98) | 0.86  (0.82 – 0.93) | 0.83  (0.79 – 0.87) | 1.00  (0.99 – 1.00) | 0.90  (0.86 – 0.93) | 0.74  (0.69 – 0.78) | 1.00  (0.99 -1.00) | 0.91  (0.87 – 0.93) | 0.75  (0.70 – 0.79) | 0.96  (0.93 – 0.98) | 0.85  (0.80 – 0.88) | 0.82  (0.78 – 0.86) |
| **Outcome: LOS** | | | | | | | | | | | | | | | |
| **MRCI** | | | | | | | | | | | | | | | |
| **LRG** | 0.58  (0.54-0.62) | 0.769  (0.734, 0.805) | 0.616  (0.575, 0.657) | 0.54  (0.48, 0.60) | 0.72  (0.68, 0.77) | 0.38  (0.33, 0.44) | 0.52  (0.47, 0.57) | 0.78  (0.73, 0.82) | 0.39  (0.34, 0.44) | 0.43  (0.38, 0.48) | 0.82  (0.77, 0.85) | 0.33  (0.28, 0.38) | 0.63  (0.57, 0.68) | 0.67  (0.62, 0.72) | 0.44  (0.39, 0.49) |
| **NB** | 0.57  (0.52, 0.61) | 0.663  (0.623, 0.703) | 0.598  (0.551, 0.644) | 0.54  (0.49, 0.59) | 0.54  (0.49, 0.58) | 0.59  (0.54, 0.63) | 0.53  (0.48, 0.58) | 0.52  (0.47, 0.58) | 0.60  (0.54, 0.66) | 0.54  (0.48, 0.59) | 0.61  (0.55, 0.66) | 0.69  (0.64, 0.73) | 0.54  (0.48, 0.59) | 0.45  (0.40, 0.51) | 0.50  (0.44, 0.55) |
| **RF** | 0.53  (0.49, 0.58) | 0.613  (0.571, 0.654) | 0.722  (0.684, 0.761) | 0.58  (0.53, 0.63) | 0.54  (0.49, 0.58) | 0.46  (0.42, 0.50) | 0.61  (0.55, 0.66) | 0.56  (0.48, 0.63) | 0.25  (0.17, 0.33) | 0.68  (0.63, 0.73) | 0.77  (0.72, 0.81) | 0.73  (0.68, 0.78) | 0.50  (0.45, 0.55) | 0.30  (0.26, 0.35) | 0.09  (0.06, 0.13) |
| **XGB** | 0.68  (0.64 – 0.71) | 0.655  (0.621, 0.690) | 0.568  (0.532, 0.604) | 0.64  (0.59 – 0.68) | 0.68  (0.63, 0.73) | 0.59  (0.53, 0.65) | 0.72  (0.67 – 0.77) | 0.63  (0.58, 0.68) | 0.55  (0.50, 0.60) | 0.76  (0.71 – 0.80) | 0.61  (0.56, 0.66) | 0.49  (0.44, 0.54) | 0.60  (0.55 – 0.65) | 0.70  (0.65, 0.75) | 0.65  (0.59, 0.70) |
| **APACHEII** | | | | | | | | | | | | | | | |

| **LRG** | 0.70  (0.66 – 0.74) | 0.76  (0.73 – 0.80) | 0.49  (0.45 – 0.53) | 0.64  (0.59 – 0.69) | 0.76  (0.71 – 0.81) | 0.57  (0.52 - 0.62) | 0.66  (0.61 – 0.71) | 0.69  (0.64 – 0.74) | 0.58  (0.53 – 0.63) | 0.63  (0.58 – 0.68) | 0.67  (0.62 – 0.72) | 0.57  (0.52 – 0.62) | 0.67  (0.62 – 0.71) | 0.78  (0.73 - 0.82) | 0.59  (0.53 – 0.64) |
| --- | --- | --- | --- | --- | --- | --- | --- | --- | --- | --- | --- | --- | --- | --- | --- |
| **NB** | 0.67  (0.63 – 0.71) | 0.72  (0.69 – 0.76) | 0.63  (0.59 – 0.67) | 0.67  (0.72 -0.72) | 0.70  (0.65 – 0.75) | 0.48  (0.43 – 0.53) | 0.67  (0.62 – 0.72) | 0.64  (0.59 – 0.69) | 0.49  (0.43 – 0.55) | 0.63  (0.58 – 0.68) | 0.62  (0.56 – 0.67) | 0.57  (0.52 – 0.62) | 0.71  (0.66 – 0.76) | 0.73  (0.68 – 0.77) | 0.40  (0.35 – 0.45) |
| **RF** | 0.65  (0.61 – 0.69) | 0.69  (0.65 – 0.73) | 0.55  (0.51 – 0.60) | 0.62  (0.57 – 0.67) | 0.66  (0.62 – 0.71) | 0.57  (0.52 – 0.62) | 0.66  (0.61 -0.71) | 0.70  (0.65 – 0.75) | 0.65  (0.59 – 0.71) | 0.66  (0.61 – 0.71) | 0.76  (0.72 – 0.81) | 0.75  (0.70 – 0.79) | 0.62  (0.56 – 0.67) | 0.59  (0.54 – 0.64) | 0.45  (0.40 – 0.50) |
| **XGB** | 0.79  (0.76 – 0.82) | 0.78  (0.75 – 0.81) | 0.82  (0.80 – 0.85) | 0.80  (0.75 -0.84) | 0.82  (0.77 – 0.86) | 0.73  (0.69 – 0.77) | 0.78  (0.73 – 0.82) | 0.74  (0.70 – 0.79) | 1.00  (0.98 – 1.00) | 0.75  (0.70 – 0.79) | 0.73  (0.68 – 0.77) | 1.00  (0.99 – 1.00) | 0.82  (0.78 – 0.86) | 0.83  (0.78 – 0.87) | 0.64  (0.59 – 0.69) |

| **MRCI&APACHEII** |
| --- |

| **LRG** | 0.70  (0.66 -0.74) | 0.76  (0.73 -0 .80) | 0.54  (0.50 -0.59) | 0.64  (0.59 -0.69) | 0.76  (0.71 – 0.81) | 0.45  (0.39 -0.50) | 0.66  (0.61 -0.71) | 0.69  (0.64 -0.74) | 0.47  (0.41 – 0.52) | 0.63  90.58 -0.68) | 0.67  (0.62 – 0.72) | 0.43  (0.38 -0.48) | 0.67  (0.62 -0.71) | 0.78  (0.73 – 0.82) | 0.48  (0.43 – 0.54) |
| --- | --- | --- | --- | --- | --- | --- | --- | --- | --- | --- | --- | --- | --- | --- | --- |
| **NB** | 0.70  (0.64 -0.72) | 0.71  (0.68 – 0.75) | 0.56  (0.52 – 0.61) | 0.66  (0.61 – 0.71) | 0.68  (0.63 -0.73) | 0.54  (0.49 – 0.59) | 0.69  (0.64 -0.74) | 0.67  (0.62 – 0.72) | 0.56  (0.50 – 0.61) | 0.68  (0.62 -0.72) | 0.70  (0.65 – 0.75) | 0.57  (0.52 – 0.62) | 0.68  (0.63 -0.73) | 0.65  (0.60 -0.70) | 0.52  (0.47 – 0.58) |
| **RF** | 0.71  (0.67 -0.75) | 0.71  (0.67 – 0.75) | 0.65  (0.61 – 0.69) | 0.56  (0.51 -0.61) | 0.65  (0.61 – 0.70) | 0.45  (0.40 – 0.49) | 0.62  (0.56 -0.67) | 0.70  (0.64 – 0.75) | 0.41  (0.35 – 0.48) | 0.66  (0.60 -0.71) | 0.76  (0.72 – 0.81) | 0.62  (0.57 – 0.67) | 0.53  (0.47 -0.58) | 0.57  (0.51 -0.62) | 0.26  (0.22 – 0.31) |
| **XGB** | 0.77  (0.74 – 0.80) | 0.82  (0.79 -0.85) | 0.76  (0.73 – 0.79) | 0.68  (0.63 – 0.73) | 0.82  (0.77 – 0.85) | 0.70  (0.66 – 0.75) | 0.70  (0.65 – 0.75) | 0.83  (0.78 -0.86) | 0.83  (0.78 – 0.87) | 0.68  (0.63 -0.73) | 0.84  (0.80 -0.880 | 0.87  (0.83 – 0.90) | 0.70  (0.65 -0.75) | 0.80  (0.75 -0.84) | 0.64  (0.59 – 0.69) |

| **SOFA** |
| --- |

| **LRG** | 0.72  (0.68 -0.76) | 0.73  (0.70 – 0.77) | 0.39  (0.35 – 0.43) | 0.63  (0.58 -0.68) | 0.64  (0.60 -0.69) | 0.49  (0.44 -0.55) | 0.71  (0.65 -0.76) | 0.71  (0.65 – 0.76) | 0.47  (0.42 -0.53) | 0.73  (0.68 -0.78) | 0.79  (0.74 – 0.83) | 0.49  (0.43 -0.54) | 0.60  (0.55 -0.65) | 0.54  (0.49 -0.60) | 0.48  (0.43 -0.54) |
| --- | --- | --- | --- | --- | --- | --- | --- | --- | --- | --- | --- | --- | --- | --- | --- |
| **NB** | 0.62  (0.58 -0.66) | 0.44  (0.40 – 0.49) | 0.77  (0.74 -0.81) | 0.60  (0.56 -0.0.65) | 0.61  (0.57 – 0.66) | 0.66  (0.61 -0.70) | 0.68  (0.62 -0.73) | 0.80  (0.73 -0.85) | 0.67  (0.62 -0.73) | 0.72  (0.66 -0.76) | 0.90  (0.86 -0.93) | 0.72  (0.67 -0.76) | 0.56  (0.51 -0.61) | 0.41  (0.36 -0.46) | 0.61  (0.56 -0.66) |
| **RF** | 0.61  (0.56 – 0.65) | 0.71  (0.67 -0.75) | 0.71  (0.67 -0.75) | 0.56  (0.52 – 0.61) | 0.70  (0.65 -0.74) | 0.71  (0.66 -0.75) | 0.66  (0.60 – 0.72) | 0.82  (0.77 -0.87) | 0.81  (0.76 – 0.86) | 0.75  (0.70 -0.79) | 0.88  (0.84 -0.91) | 0.86  (0.82 -0.89) | 0.46  (0.41 -0.51) | 0.60  (0.55 – 0.65) | 0.63  (0.58 -0.68) |
| **XGB** | 0.80  (0.77 – 0.83) | 0.83  (0.81 -0.86) | 0.91  (0.89 – 0.93) | 0.82  (0.77 -0.86) | 0.81  (0.77 -0.85) | 1.00  (0.99 – 1.00) | 0.79  (0.74 -0.83) | 0.87  (0.82 -0.90) | 0.84  (0.80 – 0.87) | 0.76  (0.71 -0.80) | 0.88  (0.85 – 0.91) | 0.82  (0.77 – 0.86) | 0.84  (0.80 -0.88) | 0.78  (0.74 – 0.83) | 1.00  (0.99 – 1.00) |

| **MRCI&SOFA** |
| --- |

| **LRG** | 0.73  (0.69 -0.76) | 0.75  (0.77 – 0.78) | 0.38  (0.34 – 0.43) | 0.63  (0.58 -0.68) | 0.64  (0.60 -0.69) | 0.49  (0.44 -0.55) | 0.67  (0.61 -0.72) | 0.71  (0.65 -0.76) | 0.47  (0.42 – 0.53) | 0.66  (0.61 -0.71) | 0.79  (0.74 – 0.83) | 0.49  (0.43 – 0.54) | 0.63  (0.58 – 0.68) | 0.54  (0.49 – 0.60) | 0.48  (0.43 – 0.54) |
| --- | --- | --- | --- | --- | --- | --- | --- | --- | --- | --- | --- | --- | --- | --- | --- |
| **NB** | 0.63  (0.59 – 0.67) | 0.59  (0.55 -0.63) | 0.72  (0.68 – 0.76) | 0.56  (0.51 -0.61) | 0.61  (0.56 – 0.65) | 0.66  (0.60 – 0.70) | 0.61  (0.55 – 0.66) | 0.68  (0.61 – 0.73) | 0.63  (0.58 – 0.68) | 0.63  (0.58 – 0.68) | 0.79  (0.74 – 0.83) | 0.63  (0.58 – 0.68) | 0.54  (0.49 -0.59) | 0.47  (0.42 – 0.52) | 0.65  (0.60 – 0.70) |
| **RF** | 0.67  (0.63 – 0.71) | 0.71  (0.67 – 0.74) | 0.57  (0.53 – 0.61) | 0.58  (0.54 -0.63) | 0.67  (0.62 -0.71) | 0.48  (0.44 – 0.52) | 0.71  (0.65 – 0.77) | 0.81  (0.76 – 0.86) | 0.32  (0.23 -0.42) | 0.80  (0.76 -0.84) | 0.88  (0.84 – 0.91) | 0.82  (0.77 – 0.86) | 0.46  (0.41 - 0.51) | 0.54  (0.49 – 0.60) | 0.09  (0.06 – 0.13) |
| **XGB** | 0.79  (0.76 – 0.82) | 0.81  (0.78 – 0.84) | 0.86  (0.83 – 0.88) | 0.79  (0.74 -0.83) | 0.77  (0.73 – 0.81) | 0.86  (0.82 -0.89) | 0.80  (0.76 -0.84) | 0.88  (0.83 – 0.91) | 0.85  (0.81 -0.89) | 0.79  (0.74 – 0.83) | 0.90  (0.87 – 0.93) | 0.86  (0.82 – 0.89) | 0.80  (0.75 – 0.84) | 0.72  (0.67 – 0.77) | 0.85  (0.81 – 0.89) |

| **Outcome: MV** |
| --- |
| **MRCI** |

| **LRG** | 0.65  (0.61, 0.69) | 0.58  (0.54, 0.62) | 0.60  (0.55, 0.64) | 0.38  (0.33, 0.42) | 0.55  (0.50, 0.61) | 0.43  (0.37, 0.48) | 0.34  (0.29, 0.40) | 0.53  (0.48, 0.58) | 0.48  (0.43, 0.53) | 0.49  (0.44, 0.55) | 0.52  (0.47, 0.57) | 0.36  (0.31, 0.42) | 0.24  (0.20, 0.29) | 0.56  (0.51, 0.61) | 0.54  (0.49, 0.59) |
| --- | --- | --- | --- | --- | --- | --- | --- | --- | --- | --- | --- | --- | --- | --- | --- |
| **NB** | 0.59  (0.45, 0.63) | 0.54  (0.50, 0.58) | 0.62  (0.58, 0.66) | 0.52  (0.47, 0.57) | 0.55  (0.49, 0.60) | 0.43  (0.38, 0.47) | 0.57  (0.52, 0.63) | 0.52  (0.47, 0.58) | 0.40  (0.34, 0.47) | 0.64  (0.58, 0.69) | 0.53  (0.48, 0.58) | 0.59  (0.54, 0.64) | 0.45  (0.40, 0.51) | 0.54  (0.48, 0.59) | 0.26  (0.21, 0.31) |
| **RF** | 0.59  (0.55, 0.63) | 0.58  (0.54, 0.63) | 0.63  (0.59, 0.67) | 0.53  (0.49, 0.57) | 0.56  (0.52, 061) | 0.55  (0.51, 0.59) | 0.65  (0.58, 0.71) | 0.67  (0.59, 0.74) | 0.81  (0.74, 0.88) | 0.79  (0.74, 0.81) | 0.85  (0.81, 0.89) | 0.93  (0.90, 0.95) | 0.36  (0.31, 0.41) | 0.31  (0.26, 0.36) | 0.30  (0.25, 0.34) |
| **XGB** | 0.75  (0.72, 0.78) | 0.79  (0.76, 0.82) | 0.74  (0.71, 0.77) | 0.78  (0.73, 0.82) | 0.78  (0.73, 0.82) | 0.74  (0.70, 0.79) | 0.72  (0.67, 0.77) | 0.80  (0.75, 0.84) | 0.74  (0.69, 0.78) | 0.72  (0.67, 0.77) | 0.82  (0.78, 0.86) | 0.76  (0.71, 0.80) | 0.78  (0.73, 0.82) | 0.75  (0.70, 0.80) | 0.72  (0.67, 0.77) |

| **APACHEII** |
| --- |

| **LRG** | 0.71  (0.67 – 0.75) | 0.75  (0.72 - 0.79) | 0.80  (0.76 – 0.83) | 0.66  (0.61 -0.71) | 0.66  (0.61 – 0.71) | 0.24  (0.20 – 0.28) | 0.68  (0.63 – 0.73) | 0.69  (0.64 – 0.75) | 0.15  (0.11 – 0.20) | 0.65  (0.60 -0.70) | 0.75  (0.71 – 0.80) | 0.26  (0.22 – 0.31) | 0.69  (0.64 – 0.740 | 0.59  (0.54 – 0.64) | 0.14  (0.10 – 0.18) |
| --- | --- | --- | --- | --- | --- | --- | --- | --- | --- | --- | --- | --- | --- | --- | --- |
| **NB** | 0.69  (0.65 – 0.73) | 0.68  (0.64 – 0.72) | 0.65  (0.61 – 0.69) | 0.60  (0.55 -0.65) | 0.62  (0.57 – 0.67) | 0.41  (0.36 – 0.46) | 0.68  (0.62 – 0.73) | 0.58  (0.53 – 0.63) | 0.33  (0.27 – 0.39) | 0.71  (0.66 – 0.76) | 0.57  (0.52 – 0.62) | 0.51  (0.45 – 0.56) | 0.56  (0.51 – 0.61) | 0.63  (0.58 – 0.68) | 0.25  (0.20 – 0.29) |
| **RF** | 0.68  (0.64 – 0.72) | 0.71  (0.67 – 0.75) | 0.61  (0.57 – 0.65) | 0.65  (0.59 – 0.70) | 0.64  (0.59 – 0.69) | 0.47  (0.42 – 0.52) | 0.66  (0.61 – 0.71) | 0.69  (0.64 – 0.75) | 0.43  (0.37 – 0.49) | 0.62  (0.57 – 0.68) | 0.77  (0.73 – 0.81) | 0.59  (0.53 – 0.64) | 0.68  (0.63 – 0.73) | 0.54  (0.49 - 0.60) | 0.32  (0.27 – 0.37) |
| **XGB** | 0.77  (0.74 – 0.80) | 0.88  (0.86 – 0.90) | 0.72  (0.68 – 0.75) | 0.75  (0.71 – 0.80) | 0.94  (0.91 – 0.96) | 0.78  (0.72 – 0.82) | 0.79  (0.74 – 0.83) | 0.83  (0.79 – 0.86) | 0.68  (0.63 – 0.72) | 0.78  (0.73 – 0.82) | 0.82  (0.77 – 0.85) | 0.60  (0.54 – 0.65) | 0.76  (0.72 – 0.81) | 0.94  (0.91 – 0.96) | 0.83  (0.79 – 0.87) |

| **MRCI&APACHEII** |
| --- |

| **LRG** | 0.71  (0.67 – 0.75) | 0.76  (0.73 – 0.80) | 0.81  (0.78 – 0.84) | 0.63  (0.58 -0.68) | 0.69  (0.64 – 0.73) | 0.24  (0.20 – 0.28) | 0.67  (0.61 -0.71) | 0.71  (0.66 – 0.76) | 0.15  (0.11 – 0.20) | 0.65  (0.60 -0.70) | 0.75  (0.71 – 0.80) | 0.26  (0.22 – 0.31) | 0.64  (0.59 – 0.69) | 0.64  (0.58 – 0.69) | 0.14  (0.10 – 0.18) |
| --- | --- | --- | --- | --- | --- | --- | --- | --- | --- | --- | --- | --- | --- | --- | --- |
| **NB** | 0.68  (0.64 – 0.72) | 0.69  (0.65 – 0.72) | 0.66  (0.62 – 0.70) | 0.62  (0.57 -0.66) | 0.59  (0.54 – 0.64) | 0.44  (0.39 – 0.49) | 0.71  (0.66 -0.76) | 0.60  (0.54 – 0.65) | 0.40  (0.35 -0.46) | 0.75  (0.71 – 0.80) | 0.68  (0.63 – 0.73) | 0.51  (0.45 – 0.56) | 0.57  (0.51 -0.62) | 0.51  (0.45 – 0.56) | 0.34  (0.29 – 0.39) |
| **RF** | 0.68  (0.64 – 0.72) | 0.76  (0.72 – 0.80) | 0.42  (0.37 – 0.46) | 0.59  (0.54 -0.63) | 0.65  (0.61 – 0.69) | 0.42  (0.38 0.46) | 0.68  (0.62 -0.73) | 0.85  (0.79 – 0.90) | 0.28  (0.22 – 0.35) | 0.73  (0.68 -0.77) | 0.92  (0.89 – 0.94) | 0.59  (0.54 – 0.64) | 0.53  (0.48 – 0.58) | 0.48  (0.43 – 0.54) | 0.17  (0.13 – 0.21) |
| **XGB** | 0.77  (0.73 – 0.80) | 0.91  (0.89 – 0.93) | 0.76  (0.74 – 0.79) | 0.76  (0.72 – 0.80) | 0.89  (0.86 – 0.92) | 0.67  (0.63 – 0.71) | 0.77  (0.72 -0.82) | 0.94  (0.90 – 0.96) | 1.00  (0.98 – 1.00) | 0.80  (0.76 -0.84) | 0.94  (0.91 – 0.96) | 1.00  (0.99 – 1.00) | 0.73  (0.68 -0.77) | 0.88  (0.84 – 0.91) | 0.52  (0.47 – 0.58) |

| **SOFA** |
| --- |

| **LRG** | 0.78  (0.75 -0.82) | 0.72  (0.69 – 0.76) | 0.92  (0.91 – 0.94) | 0.76  (0.72 -0.81) | 0.67  (0.62 – 0.71) | 0.72  (0.68 – 0.76) | 0.80  (0.75 -0.84) | 0.73  (0.67 – 0.78) | 0.81  (0.76 – 0.85) | 0.79  (0.74 -0.83) | 0.79  (0.74 – 0.83) | 0.85  (0.81 – 0.89) | 0.77  (0.73 -0.81) | 0.59  (0.53 – 0.64) | 0.65  (0.59 – 0.70) |
| --- | --- | --- | --- | --- | --- | --- | --- | --- | --- | --- | --- | --- | --- | --- | --- |
| **NB** | 0.76  (0.72 -0.79) | 0.69  (0.65 – 0.73) | 0.82  (0.79 – 0.86) | 0.76  (0.71 -0.80) | 0.64  (0.59 – 0.69) | 0.70  (0.66 – 0.74) | 0.79  (0.74 -0.83) | 0.66  (0.61 – 0.71) | 0.89  (0.84 – 0.93) | 0.78  (0.73 -0.82) | 0.72  (0.67 – 0.76) | 0.93  (0.90 – 0.96) | 0.77  (0.72 -0.81) | 0.58  (0.53 – 0.63) | 0.57  (0.52 – 0.62) |
| **RF** | 0.80  (0.77 -0.83) | 0.68  (0.64 – 0.72) | 0.90  (0.88 – 0.92) | 0.75  (0.70 -0.79) | 0.64  (0.59 – 0.68) | 0.78  (0.74 – 0.82) | 0.76  (0.71 -0.80) | 0.69  (0.64 – 0.75) | 0.88  (0.84 – 0.92) | 0.74  (0.69 -0.78) | 0.77  (0.73 – 0.81) | 0.91  (0.87 – 0.94) | 0.77  (0.72 -0.81) | 0.54  (0.49 – 0.59) | 0.73  (0.68 – 0.78) |
| **XGB** | 0.85  (0.83 -0.88) | 0.72  (0.69 – 0.76) | 0.96  (0.94 – 0.97) | 0.88  (0.83 – 0.91) | 0.71  (0.66 – 0.76) | 0.92  (0.89 – 0.92) | 0.84  (0.80 -0.87) | 0.74  (0.69 – 0.79) | 1.00  (0.99 – 1.00) | 0.81  (0.77 -0.85) | 0.77  (0.73 – 0.81) | 1.00  (0.99 – 1.00) | 0.89  (0.86 – 0.92) | 0.67  (0.62 – 0.72) | 0.91  (0.88 – 0.94) |

| **MRCI&SOFA** |
| --- |

| **LRG** | 0.79  (0.75 -0.82) | 0.73  (0.69 – 0.76) | 0.92  (0.90 – 0.94) | 0.76  (0.71 -0.80) | 0.65  (0.61 – 0.70) | 0.74  (0.70 – 0.78) | 0.79  (0.75 -0.83) | 0.69  (0.64 – 0.75) | 0.92  (0.88 – 0.95) | 0.78  (0.73 -0.82) | 0.75  (0.71 – 0.80) | 0.95  0.92 – 0.97) | 0.77  (0.73 -0.81) | 0.59  (0.53 – 0.64) | 0.65  (0.59 – 0.70) |
| --- | --- | --- | --- | --- | --- | --- | --- | --- | --- | --- | --- | --- | --- | --- | --- |
| **NB** | 0.74  (0.71 -0.78) | 0.68  (0.64 – 0.71) | 0.81  (0.78 – 0.84) | 0.71  (0.66 -0.76) | 0.59  (0.55 – 0.64) | 0.70  (0.66 – 0.74) | 0.76  (0.71 -0.80) | 0.67  (0.61 – 0.73) | 0.89  (0.84 – 0.93) | 0.75  (0.70 -0.80) | 0.80  (0.76 – 0.84) | 0.93  (0.90 – 0.96) | 0.72  (0.67 -0.77) | 0.42  (0.37 – 0.47) | 0.57  (0.52 – 0.63) |
| **RF** | 0.81  (0.78 -0.84) | 0.60  (0.56 – 0.65) | 0.86  (0.84 – 0.89) | 0.67  (0.62 -072) | 0.65  (0.60 – 0.69) | 0.64  (0.59 – 0.68) | 0.73  (0.68 -0.0.78) | 0.74  (0.68 – 0.79) | 0.82  (0.76 – 0.87) | 0.74  (0.69 -0.78) | 0.82  (0.77 – 0.85) | 0.91  (0.87 – 0.94) | 0.66  (0.61 -0.71) | 0.54  (0.49 – 0.59) | 0.45  (0.39 – 0.50) |
| **XGB** | 0.87  (0.85 – 0.90) | 0.80  (0.77 – 0.83) | 0.93  (0.91 – 0.95) | 0.90  (0.86 -0.93) | 0.76  (0.72 – 0.80) | 0.89  (0.85 – 0.92) | 0.86  (0.82 -0.89) | 0.84  (0.80 – 0.88) | 1.00  (0.99 – 1.00) | 0.84  (0.79 – 0.87) | 0.87  (0.83 – 0.90) | 1.00  (0.99 – 1.00) | 0.91  (0.88 – 0.94) | 0.72  (0.67 – 0.77) | 0.86  (0.82 -0.90) |

**Figure S11: Variable importance for inpatient mortality derived from machine learning algorithms**





**Figure S12: SHAP plot for predictors of inpatient mortality across the general population (only XGBoost algorithm)**

**
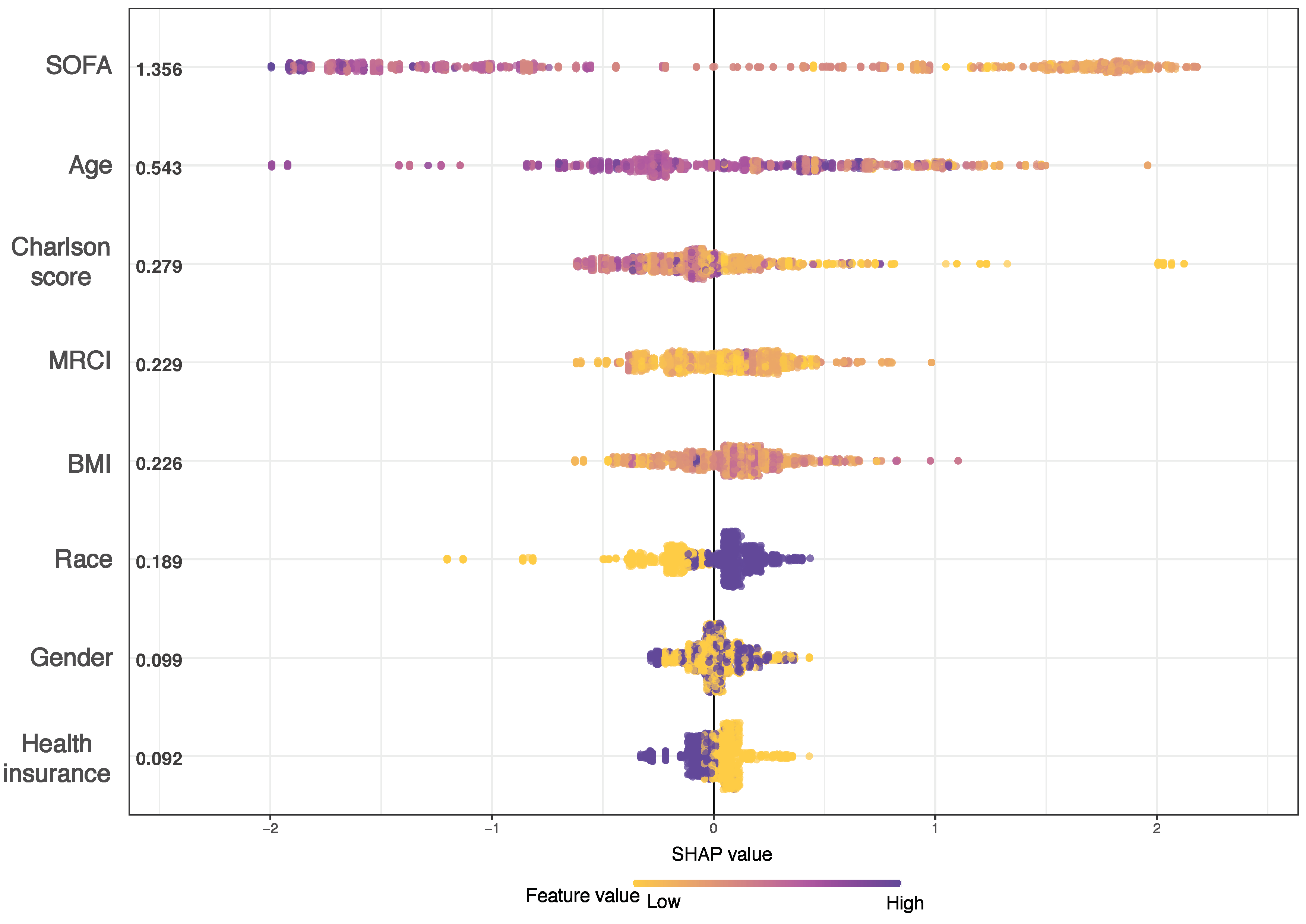
**

**Figure S13: Variable importance for inpatient LOS derived from machine learning algorithms**

**

**

**Figure S14: SHAP plot for predictors of inpatient LOS across the general population (only XGBoost algorithm)**

**
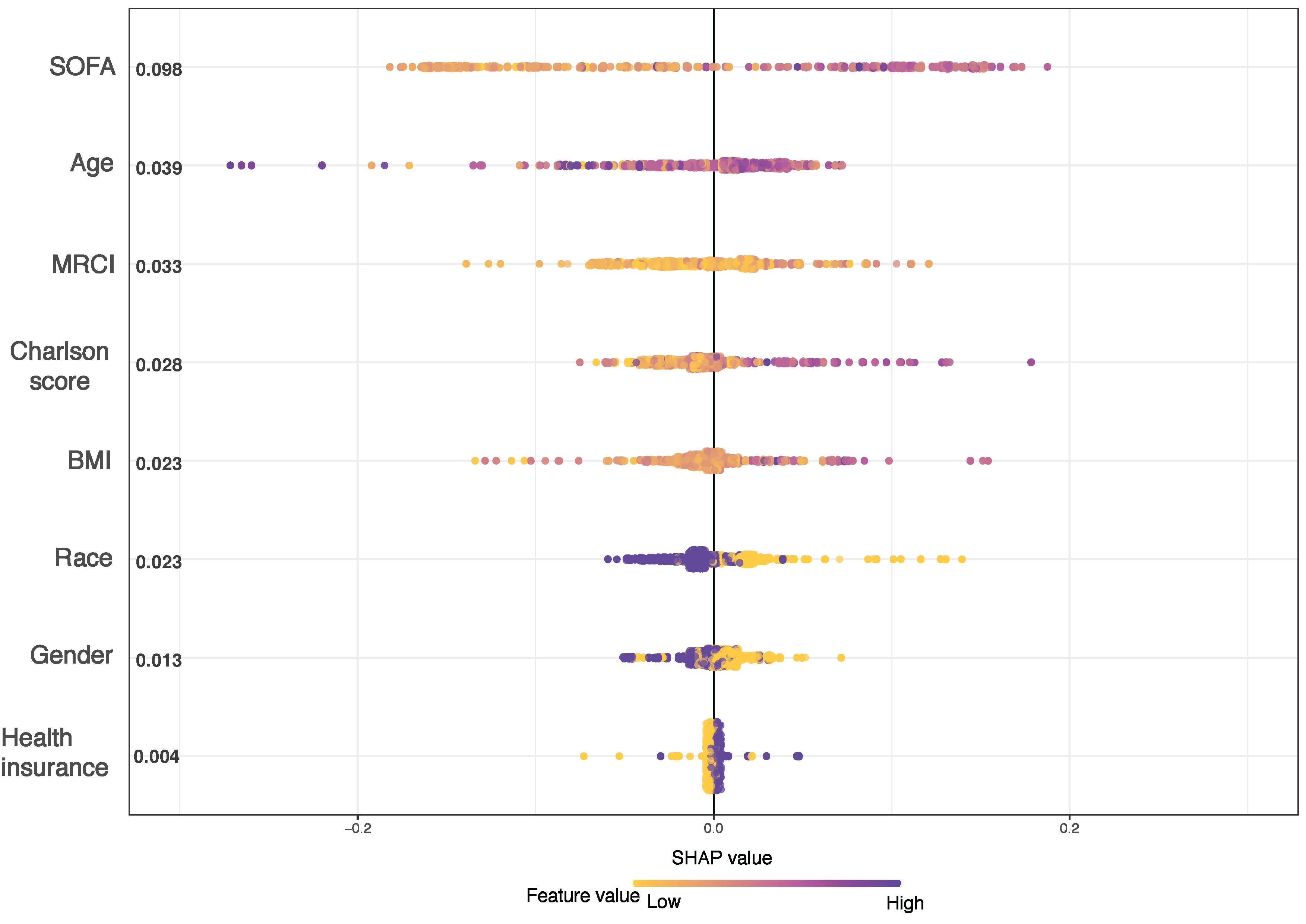
**

**Figure S15: Variable importance for inpatient MV derived from machine learning algorithms**

**

**

**Figure S16: SHAP plot for predictors of inpatient MV across the general population (only XGBoost algorithm)**

**
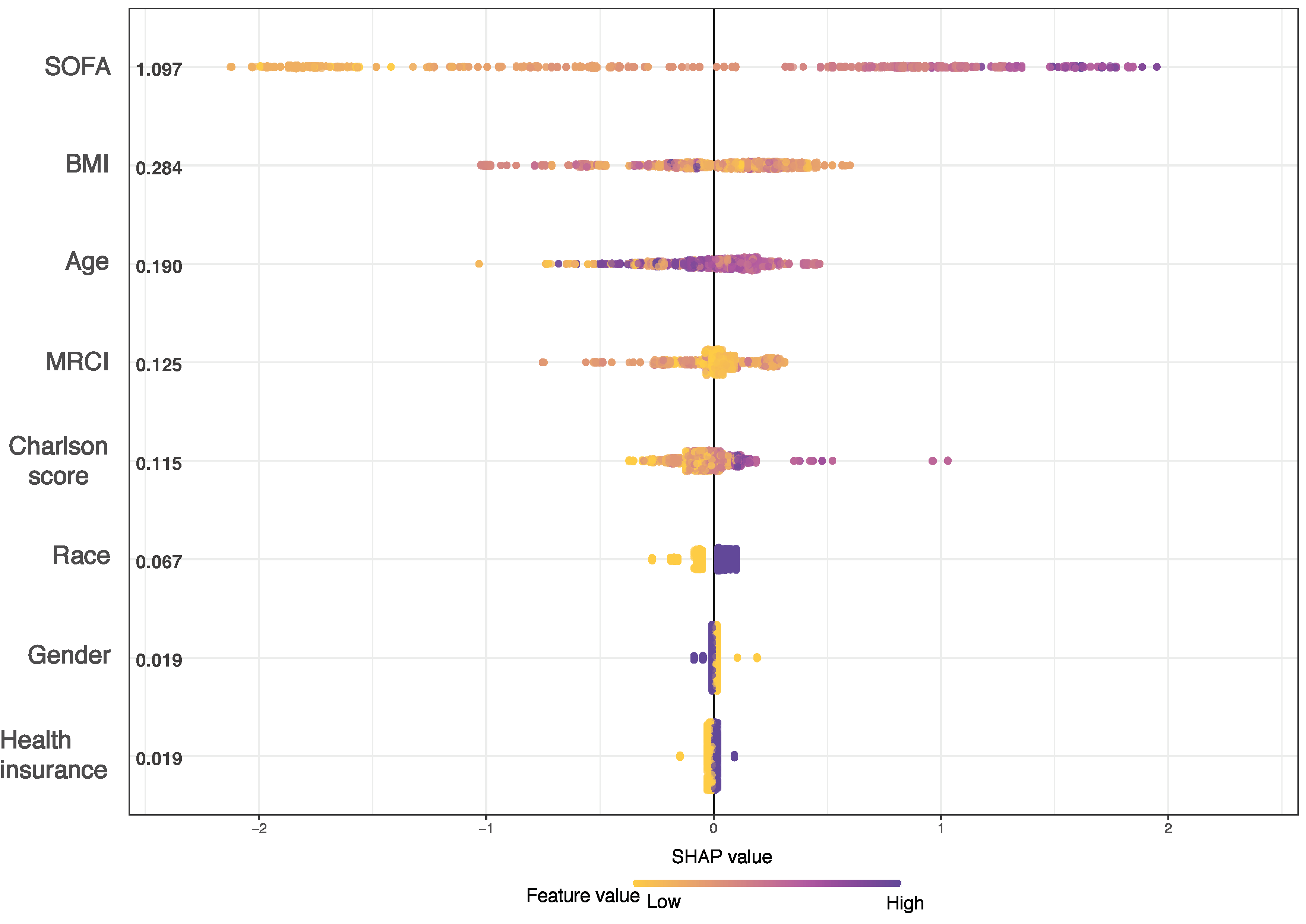
**
